# Moderating effect of people-oriented public health services on depression among people under mandatory social isolation during the COVID-19 pandemic: a cross-sectional study in China

**DOI:** 10.1186/s12889-021-11457-6

**Published:** 2021-07-12

**Authors:** Bolin Cao, Dongya Wang, Yifan Wang, Brian J. Hall, Nan Wu, Meimei Wu, Qishan Ma, Joseph D. Tucker, Xing Lv

**Affiliations:** 1 School of Media and Communication, Shenzhen University, Shenzhen, People’s Republic of China; 2 Global and Community Mental Health Research Group, Department of Psychology, University of Macau, Macao, SAR People’s Republic of China; 3 Health, Behavior and Society, Johns Hopkins Bloomberg School of Public Health, Baltimore, USA; 4 Shenzhen Center for Disease Control and Prevention, Shenzhen, Guangdong Province People’s Republic of China; 5 University North Carolina at Chapel Hill, Project-China, Guangzhou, China; 6 Faculty of Infectious and Tropical Diseases, London School of Hygiene and Tropical Medicine, London, UK

**Keywords:** COVID-19, Coronavirus, Depression, Mental health, Public health services

## Abstract

**Background:**

Public health measures, such as social isolation, are vital to control the spread of the coronavirus disease 2019 (COVID-19), but such measures may increase the risk of depression. Thus, this study examines the influencing and moderating factors of depressive symptoms among individuals subjected to mandatory social isolation.

**Methods:**

An online cross-sectional survey was conducted to collect data from people under mandatory home or centralized social isolation in Shenzhen, China, from February 28 to March 6, 2020. The perceived risk of infection with COVID-19, perceived tone of media coverage, perceived quality of people-oriented public health services, and their depressive symptoms were assessed. Three rounds of stepwise multiple regression were performed to examine the moderating effects after controlling various variables, such as demographics, duration and venue of mandatory social isolation, infection and isolation status of family, time spent on COVID-related news, and online social support.

**Results:**

Among the 340 participants, 57.6% were men, the average age was 35.5 years old (SD = 8.37), and 55.6% held a bachelor’s degree or above. Individuals subjected to mandatory social isolation generally reported low levels of depressive symptoms. Perceived susceptibility to infection was relatively low, whereas perceived tone of media coverage was mainly positive. In terms of perceived quality of public health services, 12 (3.5%), 103 (30.3%), and 225 (66.2%) participants reported low, medium, and high quality of people-oriented services, respectively. Perceived susceptibility was positively associated with depression, whereas perceived tone of media coverage was negatively associated. The quality of people-centered public health services moderated the association between perceived risk and depressive symptoms and between perceived tone of media coverage and depressive symptoms.

**Conclusions:**

This study revealed the depressive symptoms among individuals subjected to mandatory social isolation during the COVID-19 pandemic and highlighted that frontline public health workers play a critical role in protecting public mental health.

**Supplementary Information:**

The online version contains supplementary material available at 10.1186/s12889-021-11457-6.

## Background

The coronavirus disease 2019 (COVID-19) first broke out in Wuhan, China, in December 2019, which has since affected more than 200 countries. According to the data of the World Health Organization (WHO), 75 million cases and 1.6 million deaths due to COVID-19 have been confirmed worldwide as of December 2020 [1]. Social isolation has been the cornerstone of effective public response to this health crisis [2] because this method can prevent transmission to close contacts and other people. By late January, several cities in China have begun to implement stringent social isolation policies. Then, by the middle of February 2020, such policies have restricted the movement of more than 500 million persons across 80 Chinese cities [3].

Although evidence that mass quarantine is an effective preventive measure has been provided, scholars have called for caution in implementing social isolation due to its adverse effects on mental health [4]. Such mental health problems include, but not limited to, anxiety disorder and depression. These problems occur mainly because of fear of infection, distress and boredom, reduced social and physical contact, and disruption of normal life during social isolation [5]. Previous research showed that the negative effects of social isolation may continue up to 3 years after the isolation period, which is similar to the influence of post-traumatic stress disorder [6]. Among the various psychological problems, depression is the most common mental health issue related to COVID-19 [7].

Several factors could influence depression among individuals subjected to mandatory isolation. First, perceived threat and risk of public health emergencies, including perceived susceptibility and severity, may influence depression levels among such individuals [8]. Perceived susceptibility refers to the likelihood that people feel that they will be infected, whereas perceived severity indicates that people consider the consequences of infection as fatal [9]. High levels of perceived risk may be associated with increased depressive symptoms [10]. Second, the media can provide many channels for spreading news and information about the COVID-19 pandemic and forming one’s understanding of such information. However, differences of stances and opinions across these media channels may exist, and the tone of media reports may range from positive and encouraging (i.e., medical staff exerting heroic efforts on the front line in the fight against the COVID-19 pandemic) to negative and desperate (i.e., severe shortage of personal protective equipment for healthcare workers). People subjected to mandatory social isolation who are affected by negative media reports are more likely to suffer from mental health problems [11].

The existing research rarely mentions the impact of public health services on the mental health of people subjected to mandatory isolation. During the COVID-19 pandemic, China was the first country to implement social distancing policies, which were supported by multiple public health services. Community-based public health service providers are responsible for providing daily support to individuals under mandatory isolation. The main public health services include delivery of daily meals, provision of essentials, monitoring of body temperature, and provision of primary guidelines for seeking medical help as necessary [12]. Frontline public health workers providing these services were deeply involved in the lives of people under mandatory social isolation and thus may play a vital role in their mental health. The provision of high-quality people-oriented public health services may alleviate the depressive symptoms of people under mandatory social isolation, whereas low-quality community-based public health services may exacerbate their depressive symptoms.

In addition, the quality of public health services procured may moderate the associations between perceived risk and perceived tone of media coverage and depressive symptoms among individuals under mandatory social isolation. In line with the theory of people-centered public health services, public health workers are encouraged to show empathy, respect, engagement, individualized focus, and coordinated care to enable people to live a meaningful life [13]. Calling for humanism in medicine and holistic healing has been a longstanding principle [14]. This principle aims to place human beings at the center, express sympathy to those suffering, and promote better an understanding and experience of medical services. In this manner, people-oriented public health services could play salient roles in improving mental health among people subjected to mandatory social isolation. For individuals with high-risk perception, high-quality people-oriented public health services may provide a sense of comfort and security, which are associated with less depressive symptoms. By contrast, low-quality public health services may aggravate negative feelings and are associated with increased depressive symptoms. Similarly, high-quality people-oriented public health services could alleviate negative feelings among individuals who perceive a negative tone from media reports, which is related to less depressive symptoms. Moreover, high-quality people-oriented public health services can enhance the positive perception of media coverage. Thus, such services would be associated with less depressive symptoms among individuals subjected to mandatory social isolation.

The objective of this study is threefold: (1) to examine depression among the isolated population, (2) to investigate the association among perceived risk, perceived tone of media reports, and depression during COVID-19, and (3) to explore the moderating role of people-centered public health services on the association between perceived risk and tone of media reports on depressive symptoms during the COVID-19 pandemic.

## Methods

### Research context

Considering the rapid spread of COVID-19, many provincial and municipal Chinese governments have taken the highest level of response to major public health emergencies since the end of January 2020 [15]. These responses included strict measures in conducting a comprehensive screening of people arriving from cities with a high disease burden. Shenzhen City in Guangdong Province is one of the cities implementing such strict response measures. The city is home to 4 million permanent residents and a floating population of 8.5 million. All residents who visited or stayed in key pandemic areas (e.g., Hubei Province) in the past 14 days before returning to Shenzhen were required to undergo 14 days of mandatory social isolation. Individuals who have been in close contact with patients diagnosed positive for COVID-19 were also required to undergo stringent quarantine. As of February 28, 2020, 418 positive cases of COVID-19 were confirmed in Shenzhen. Among them, 141 were from Hubei Province, which was the hardest hit area, and 261 cases were local [16].

Unlike many countries that encouraged citizens to self-isolate, China implemented mandatory social isolation for these groups who visited or stayed in key pandemic areas from late January to late April of 2020. As of February 21, 2020, the number of residents in Shenzhen who were under home or centralized social isolation reached approximately 25,000. According to the regulations in Shenzhen, the difference between home-based and centralized isolation is whether the person required for mandatory social isolation has the conditions for home isolation, such as whether each home for isolation has a single room and independent bathroom and whether community management is available for the place. If the home does not meet the conditions for home-based mandatory social isolation, the individual needs to be isolated in a hotel. Regardless of home-based or centralized isolation, public health worker’s services were not different. However, people may feel more familiar and comfortable with the home environment.

### Sample collection

The study conducted an online cross-sectional survey among individuals subjected to mandatory home or centralized social isolation in Shenzhen from February 28 to March 6, 2020. To be eligible for the study, participants must be above 16 years old and currently under or have experienced and completed mandatory home or centralized social isolation during the survey period. At the time of the survey, conducting a field investigation was not possible or allowed. Thus, this study conducted online surveys as this method provides a unique opportunity for research in the COVID-19 era and is the tool of choice among researchers [17].

First, out of 10 districts in Shenzhen, two districts, namely, Luohu and Longgang, were randomly selected for investigation. Luohu District is located in the south part of Shenzhen, which is very close to Hong Kong with a well-developed economy. Longgang District lies in the north part of Shenzhen, which is far from Shenzhen’s Bay Area with a relatively underdeveloped economy. The geographical area of Longgang District is 388.22 m^2^, which is much larger than that of Luohu District (78.75 m^2^). Both districts are composed of a large number of permanent and floating populations. As of the end of 2018, Luohu District had a total population of 1.03 million and a permanent population of 0.6 million. The permanent population of Longgang District exceeded 2.3 million, and of which, the permanent population was only 0.72 million [18]. Second, to reach the individuals subjected to mandatory social isolation who were considered a part of the “hidden population” [19, 20], the researchers cooperated with public health workers in local communities to distribute the online questionnaire. The survey questionnaire was initially published in the working groups of the two districts for pandemic management through WeChat (China’s most popular social media platform). A total of 67 public health service workers in the local communities have seen the survey link and forwarded the questionnaire to individuals subjected to mandatory social isolation.

Specifically, as public health service workers were responsible for providing daily necessities and monitoring health status (i.e., body temperature), they had access to the contact information of individuals under mandatory isolation. Moreover, WeChat is easy to use and can transmit pictures to facilitate monitoring. Thus, public health workers and people under mandatory social isolation tend to be friends on WeChat. The local public health workers sent the survey links or quick response codes containing the survey link to the participants through WeChat. The participants read the informed consent before answering the survey and then voluntarily and anonymously completed the survey. The majority of participants spent 3–5 min to fill the questionnaire (Additional file 1). Because the online survey tool reminded respondents to answer all the required questions, the study contained no missing data. Overtly incorrect and unreasonable entries, however, were removed and considered as missing data. According to backstage data of the survey link, 65 people did not complete the questionnaire after entering the survey’s webpage. The response rate was 84%.

The sample size was calculated based on the assumption that people under home or centralized social isolation would present 10% more depressive symptoms than regular individuals during the COVID-19 pandemic [21]. Therefore, a sample size of 335 would ensure that statistical analysis with α = .05 has an 80% ability to examine the differences in depressive symptoms of people under mandatory social isolation.

The study obtained approval from the institutional review board of the Shenzhen Center for Disease Control and Prevention.

### Measurements

#### Depression

Depression refers to various negative psychological symptoms, such as depressive mood, loss of interest, fatigue, difficulty in paying attention, and suicidal ideation. Depression was measured using the Patient Health Questionnaire Depression scale (PHQ-9) [22], which was previously validated for use among Chinese adults. The participants rated the extent to which they experienced nine psychological symptoms using a four-point scale, ranging from 0 = “not at all” to 3 = “nearly every day.” The reliability of the scale as used in the study was acceptable with a Cronbach’s alpha coefficient of .89. The scores of the nine items were summed for analysis. Referring to other studies using PHQ-9, this study also considered 0–4 points as no depression, 5–9 points as mild depression, 10–14 points as moderate depression, 15–19 points as moderately severe depression, and 20–27 points as a severe depression [22].

#### Perceived risk

Perceived risk indicates individuals’ subjective perception of certain risks, which are specifically represented by perceived severity and perceived susceptibility. In the present study, perceived severity was assessed by the perception of how long the pandemic would continue to influence people’s life. The participants rated their responses using a five-point scale, ranging from 1 = “Less than 1 week” to 5 = “More than 6 months.” In addition, perceived susceptibility was measured by the perception of the possibility that one could contract the virus during social isolation. The participants rated the probabilities from 1 = “highly unlikely” to 5 = “highly likely.”

#### Perceived tone of media coverage

The perceived tone of media coverage showcases the tone or emotions perceived from the media coverage on COVID-19. The perceived tone of media coverage was evaluated using seven items on a bipolar semantic scale. The seven pairs of opposite adjectives included negative versus positive, critical versus encouraging, complaining versus forgiving, nonreflective versus reflective, worried versus composed, indifferent versus touching, and timid versus brave. The participants rated their responses using a seven-point scale, ranging from − 3 to 3. The Cronbach’s alpha coefficient reached .91, which indicated excellent reliability. The scores of the seven items were averaged for analysis.

#### People-oriented public health services

People-oriented public health services measured whether public health officials and workers that were designated to support the daily life routine of people under mandatory home or centralized social isolation were understanding, caring, and trustworthy. In total, three statements were presented, namely, “Public health service workers responded to my question in ways that I can understand,” “Public health service workers cared about my feelings and emotions,” and “I perceived the public health service workers as trustworthy.” The participants rated their responses using a five-point scale, ranging from 1 = “Strongly disagree” to 5 = “Strongly agree.” Reliability was considered excellent with a Cronbach’s alpha coefficient of .95. The scores for each item were averaged and further categorized people-oriented public health services into high (average scores higher than 4), medium (average scores between 3 and 4) and low (average scores below 3) quality.

#### Control variables

The study controlled for demographic variables, such as age (continuous variable), sex (female = 0; male = 1), level of education (categorical variable from primary education to master’s degree or above), and monthly income (categorical variable from 0 to more than 30,000 RMB). Previous studies found that media exposure and online social support could influence depressive symptoms [23, 24]. Therefore, other factors such as participants’ time spent on COVID-related news and social support received online were controlled for in the analysis [25]. Time spent on COVID-related news was measured as the time that the participants spent paying attention to COVID-related news, which ranges from a few (less than 1 h) to many (more than 7 h) times per day. Online social support was measured through participant reports on information, emotional, instrumental, and esteem support received from others online. Using a five-point scale, the participants rated whether statements, such as “When I feel scared, I turn online to my relatives and/or friends to talk about my feelings,” were similar to their experiences.

In addition, the duration of social isolation, venue of social isolation, infection status of family members, isolation status of family members, and separation from family members/friends during isolation were also used as control variables. The duration of social isolation pertained to the start and end dates of social isolation. If the respondent was isolated during the survey, then the duration was measured by subtracting the start date from the date of participation in the survey. The venue of social isolation was measured by whether the participants were isolated at home or a hotel. The infection status of family members was measured by whether the participant has family members confirmed to be infected with COVID-19 virus. Moreover, the isolation status of family members was measured by whether the participant had family members who were under mandatory social isolation. Finally, the participants reported whether they lived with family members/friends or they were with family members during the mandatory social isolation process to measure separation state from family/friends during isolation.

### Statistical analysis

The study first described the demographic characteristics, risk perceptions, and depressive symptoms of the participants. Multiple regression was then conducted to examine the main effects of perceptions of susceptibility, severity, tone of media coverage, and people-oriented public health services on depression. These variables were mean-centered to avoid multicollinearity before conducting moderation analyses. Finally, three rounds of stepwise multiple regression were performed to examine the moderating effects, which can clearly determine the effects of various factors and those of the interaction terms [26]. In the stepwise regressions, the first, second, and third layers mainly included control variables, main research factors, and interactive items, respectively. Three sets of interaction terms, namely, interactions between perceived susceptibility and people-centered public health services; perceived severity and people-centered public health services; perceived tone of media and people-centered public health services, were independently added to the model.

Age, sex, education, monthly income, time spent on COVID-related news, duration of social isolation, venue of social isolation, infection status of family members, isolation status of family members, status of separation from family/friends during isolation, and online social support were controlled for in all regression models. In case of missing data, since the missing data were random and caused by input errors, we retained the cases if the missing values were not the primary exposure or outcome variables. We transformed the missing data and applied the mean replacement method for further analysis and modeling [27]. Data analyses were performed using SPSS Statistics (IBM SPSS version 26.0). Statistical significance was set at *p*-values less than or equal to .05.

## Results

### Characteristics of the participants

Among the 340 participants under mandatory social isolation, 196 (57.65%) were male. The average age was 35.51 years (SD = 8.37), which ranged from 17 to 68 years, and more than half (55.58%) held a bachelor’s degree or above. Moreover, the monthly income for the majority of the participants (57.35%) was more than 1100 USD (8000 RMB). Table 1 provides a list of the demographic characteristics.

**Table 1:** Characteristics and behaviors of individuals under mandatory social isolation in Shenzhen (*N* = 340)

Variable	Characteristics	N	%
Age	M = 35.51, SD = 8.37		
Sex	Male	196	57.65
	Female	144	42.35
Education	Junior high school or below	34	10.00
	High school	117	34.41
	Undergraduate	175	51.47
	Master or above	14	4.11
Monthly income	No income	31	9.12
	Lower than 5000 RMB	33	9.71
	5000–8000 RMB	81	23.82
	8001–12,000 RMB	81	23.82
	12,001–30,000 RMB	87	25.59
	Higher than 30,000 RMB	27	7.94
Time on COVID-related news	Less than one hour	30	8.82
	1–2 h	138	40.59
	3–4 h	124	36.47
	5–6 h	33	9.71
	More than 7 h	15	4.41
Duration of social isolation ^a^	M = 10.52, SD = 6.56		
Venue of social isolation	Centralized (Hotel)	100	29.41
	Home	293	86.18
	Both	57	16.76
	Other	4	1.18
Infection status of family members	Family members are uninfected	338	99.41
	Family member has been confirmed to be infected	2	0.59
Isolation status of family members	Family members have not been mandatorily isolated	213	62.65
	Family member has been or is being mandatorily isolated	127	37.35
Status of separation from family/ friends during isolation	Live with family/ friends during isolation	154	45.29
	Live away from family/ friends during isolation	136	40.00
	Other	50	14.71
Online social support	M = 2.83, SD = 0.74		

^a^ Note: Six (1.76%) participants inserted unreasonable dates for the start date of mandatory social isolation, so their duration of mandatory social isolation could not be calculated and was considered as missing data. The mean was used to replace these missing data for further analysis

The participants scored an average of 2.19 (SD = 3.57) in the depression scale, which indicates generally low levels of depression. In this study, a total of 284 (83.53%) participants reported no depression, whereas 41 (12.06%), 11 (3.24%), 2 (.58%), and 2 (.58%) pointed to mild, moderate, moderately severe, and severe levels of depression, respectively. Furthermore, 12 (3.53%), 103 (30.29%), and 225 (66.18%) of the participants indicated the quality of people-oriented public health services as low, medium, and high, respectively. Regarding risk perception, the average score for perceived severity was 2.55 (SD = .87), and the average score for perceived susceptibility was 1.36 (SD = .54). Perceived tone of media coverage was mainly positive (M = 1.97, SD = 1.05). Finally, the average score for online social support was 2.83 (SD = .74).

### Main effects on depressive symptoms

Perceived susceptibility was significantly associated with depressive symptoms (b = 1.04, *p* < .01), whereas the opposite is true for perceived severity (b = .16, *ns*). The results indicate that individuals under mandatory social isolation who perceived a high possibility of becoming infected were more likely to report depressive symptoms. In other words, a 1-unit change in perceived susceptibility was associated with a 1.04-unit increase in depressive symptoms. In addition, the perceived tone of media coverage was negatively associated with depressive symptoms (b = −.46, *p* < .05), which indicated that a positively perceived media tone was related to less depressive symptoms. That is, a 1-unit increase in perceived positive media tone was associated with a .46-unit decrease in depressive symptoms. Moreover, the results for regression demonstrated that people-oriented public health services were not directly correlated with depression (b = −.60, *ns*). The adjusted R-square of the main model was .065, which indicated that these factors could explain 6.5% of depressive symptoms.

### Interaction effects on depression

**Fig. 1:**
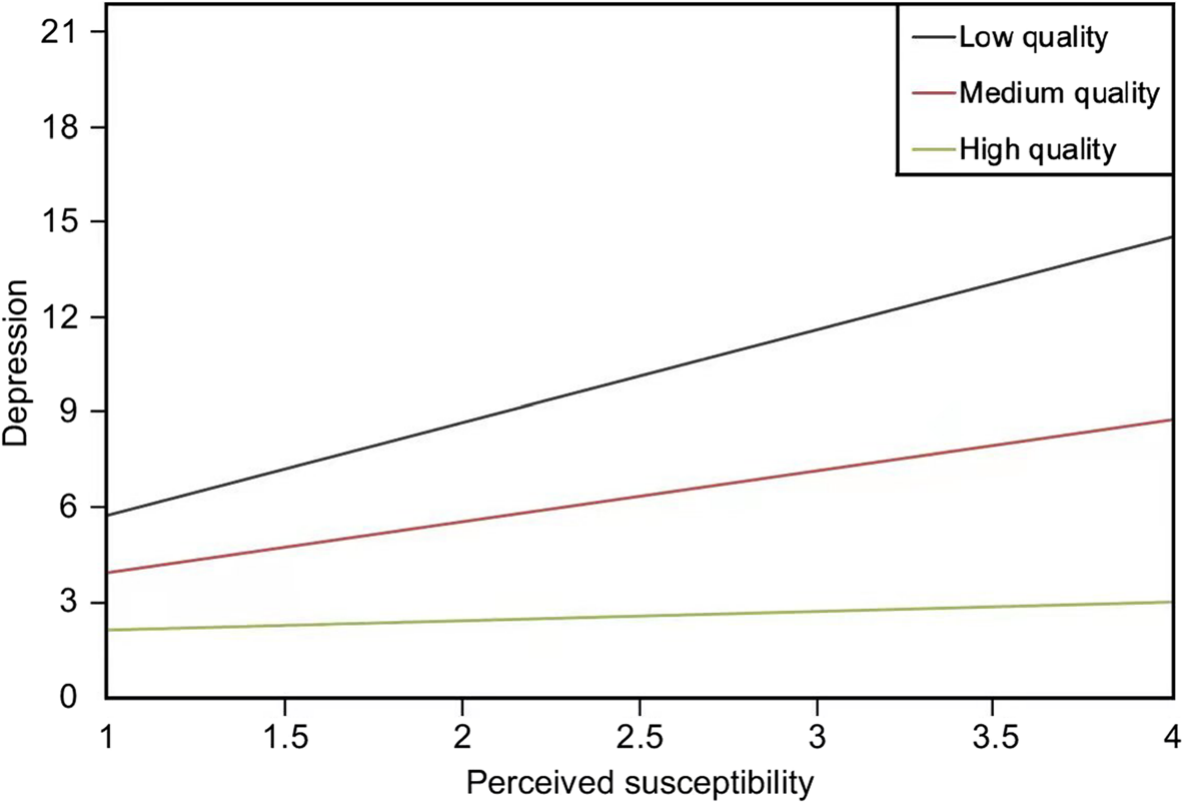
The effects of interaction between perceived susceptibility and people-oriented public health services on depressive symptoms

**Fig. 2:**
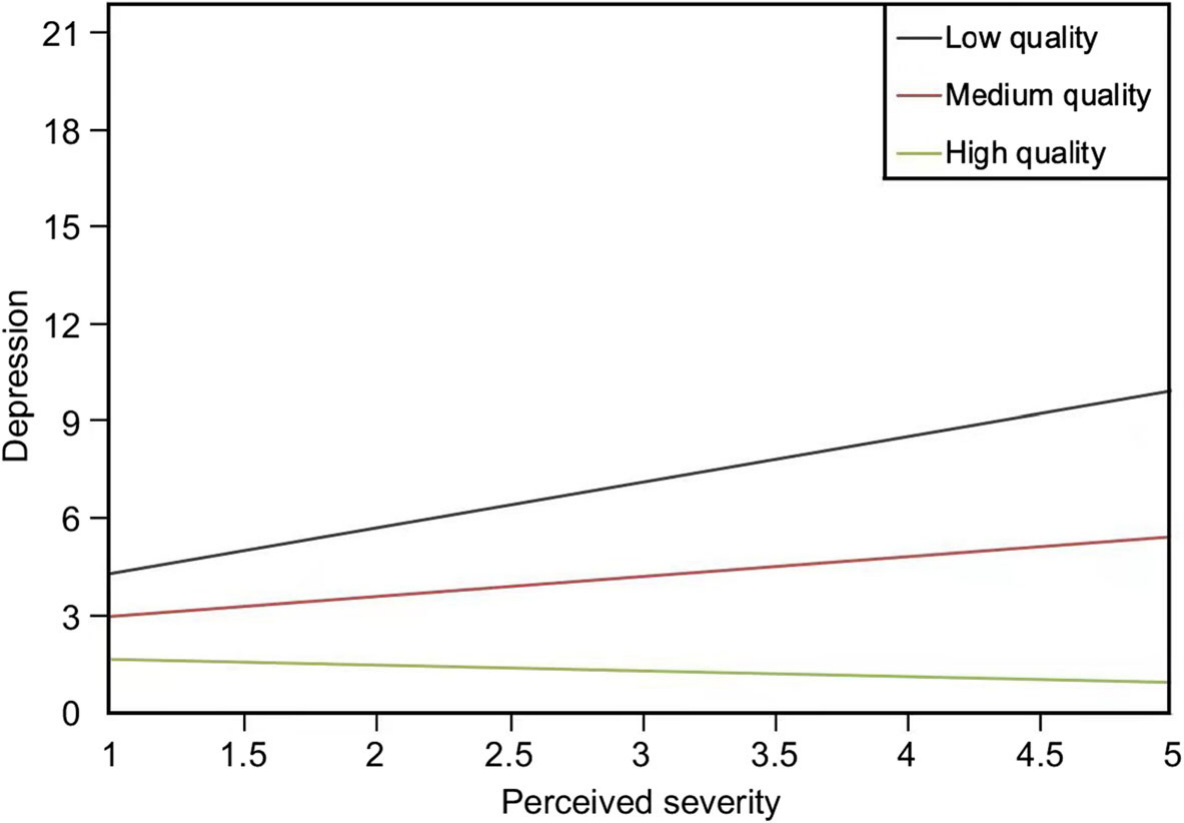
The effects of interaction between perceived severity and people-oriented public health services on depressive symptoms

Model 2 in Table 2 indicates that the interaction term between perceived susceptibility and people-oriented public health services was significant, with the model’s explanatory power increased to 8.1% in adjusted R-square. The interaction between perceived susceptibility and people-oriented public health services exerted a significant effect on depressive symptoms (b = − 1.33, *p* < .05)*.* In other words, a 1-unit increase in the quality of people-oriented public health services corresponded to a 1.33-unit decrease in the positive effect of perceived susceptibility on depressive symptoms. For socially isolated individuals suspected of having COVID-19, the high quality of people-oriented public health services attenuated depressive symptoms (Fig. 1). The study found that the interaction term between perceived severity and quality of public health services was also significant, with the model’s adjusted R-square increased to 7.4%. The interaction between perceived severity and people-oriented public health services exerted a significant effect on depressive symptoms (b = −.79, *p* < .05)*.* That is, a 1-unit increase in the quality of people-oriented public health services corresponded to a .79-unit decrease in the positive effect of perceived severity on depressive symptoms (Fig. 2). Furthermore, for socially isolated individuals who perceived the COVID-19 pandemic as severe and fatal, the high quality of people-oriented public health services alleviated depressive symptoms.

**Table 2:** Main and interaction effects on depression (*N* = 340)

	Model 1		Model 2		Model 3		Model 4	
	b	*p*	b	*p*	b	*p*	b	*p*
Intercept	2.46	<.001***	2.27	<.001***	2.39	<.001***	2.28	<.001***
**Main effects**								
Perceived susceptibility (PSU)	1.04	.005**	1.63	<.001***	.99	.008**	.95	.01*
Perceived severity (PSE)	.16	.47	.16	.48	.62	.05	.18	.41
Perceived tones of media coverage (PTM)	−.46	.02*	−.43	.03*	−.46	.02*	−.96	<.001***
People-oriented public health services (PPH)	−.60	.11	−.47	.20	−.54	.14	−.40	.29
**Moderating effects**								
PSU *PPH			−1.33	.01*				
PSE *PPH					−.79	.04*		
PTM *PPH							.87	.006**
**Covariates**								
Age	−.04	.09	−.04	.14	−.04	.09	−.04	.08
Sex	.54	.19	.53	.19	.54	.19	.46	.26
Education	−.03	.91	.02	.94	.00	.99	−.03	.92
Monthly income	.08	.60	.07	.67	.08	.62	.08	.58
Time spent on news	−.02	.91	−.07	.75	−.01	.96	.01	.95
Time of Isolation	−.02	.56	−.02	.51	−.02	.47	−.03	.43
Venues of social isolation	.24	.60	.34	.45	.19	.67	.28	.52
Infection status of family	5.64	.03*	5.78	.02*	5.65	.02*	5.51	.03*
Isolation status of family	−.04	.93	−.01	.99	−.09	.84	−.09	.84
Status of separation from family/ friends	−.62	.16	−.63	.15	−.54	.22	−.65	.14
Online social support	.42	.13	.41	.13	.39	.15	.38	.16
Adjusted R square	.065		.081		.074		.084	

Note: Model 1 examined the main effects of perceived susceptibility, perceived severity, perceived tones of media coverage, and people-oriented public health services on depression. Model 2 examined the effects of the interaction between perceived susceptibility and people-oriented public health services on depressive symptoms. Model 3 examined the effects of the interaction between perceived severity and people-oriented public health services on depression. Model 4 assessed the effects of the interaction between perceived tone of media coverage and people-oriented public health services on depressive symptoms

**Fig. 3:**
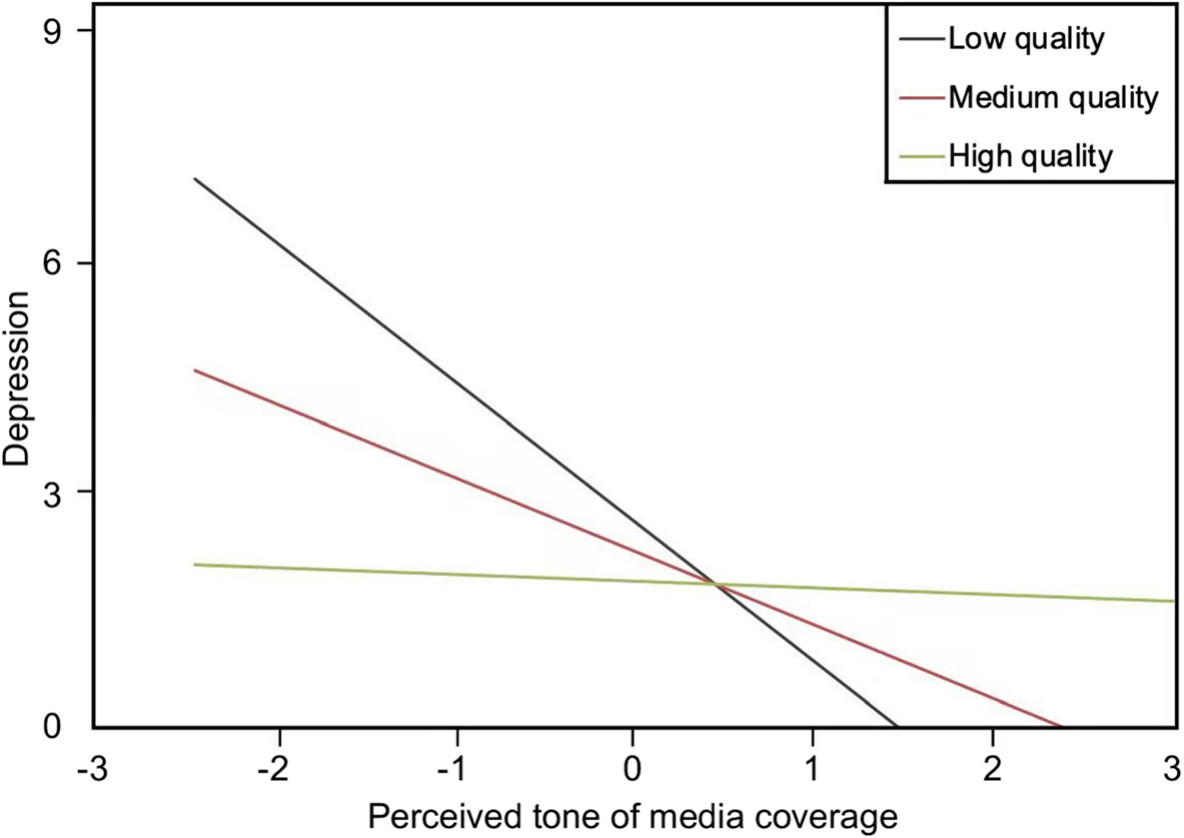
The effects of interaction between perceived tone of media coverage and people oriented public health services on depressive symptoms

The interaction term between perceived tone of media coverage and people-oriented public health services was significant, with an 8.4% increase in adjusted R-square. In addition, the interaction between perceived tone of media coverage and people-oriented public health services yielded a significant effect on depressive symptoms (b = .87, *p* < .01). Figure 3 illustrates that the low quality of people-oriented public health services was associated with increased depressive symptoms among individuals subjected to social isolation who perceived a negative tone in media coverage. That is, a 1-unit increase in the quality of people-oriented public health services was related to a .87-unit decrease in the positive effect of a perceived negative tone of media on depressive symptoms. However, for participants who perceived a positive tone in media coverage, the high quality of people-oriented public health services was associated with less depressive symptoms. An additional unit of quality of people-oriented public health services was related to a .87-unit increase in the negative effect of perceived positive tone of media coverage on depressive symptoms.

## Discussion

Public health measures, such as social isolation, physical distancing, and quarantine, are widely considered an essential part of controlling the spread of COVID-19. This study addressed the possible mental health problems among people subjected to mandatory home or centralized social isolation. The study results indicated that perceived susceptibility to infection and perceived tone of media reports were associated with depressive symptoms. Additionally, the quality of people-oriented public health services negatively moderated the associations among perceived susceptibility, perceived severity, and depressive symptoms. However, such a quality positively moderated the association between perceived tone of media coverage and depressive symptoms. The study emphasized the importance of people-oriented public health services during mandatory social isolation and extended scholarly understanding of the interaction among risk perception, the role of media reports, and the quality of public health services.

People subjected to mandatory isolation generally reported mild symptoms of depression. In the study, the levels of depression of the participants were relatively low compared with those of other research findings during the Ebola and SARS outbreaks [28]. Specifically, Tang et al. [20] conducted a study on quarantined populations during the COVID-19 pandemic in China. They found that people subjected to mandatory isolation in unaffected areas (i.e., all areas except Wuhan) were more depressed compared with those in affected areas (i.e., Wuhan) and those not quarantined. They further explained that the stringent screening conducted in unaffected areas among residents from Wuhan resulted in extreme depression and anxiety. However, our study revealed an entirely different scenario because the majority of our participants were not depressed. In fact, their levels of depression were lower than those during non-emergency periods [20, 29]. This result can be due to many reasons. First, although people were subjected to mandatory social isolation, their mentality was likely relatively relaxed because they had moved into an unaffected area. Second, most participants subjected to mandatory social isolation stated in the survey that they believed that the pandemic would be brought under control in less than half a year. Moreover, according to the WHO, COVID-19 is more deadly for the elderly population and those with chronic illnesses and/or poor health conditions compared with other diseases [30]. This news and information may have alleviated depression among people subjected to mandatory social isolation. Third, Shenzhen’s well-known service quality and efficient government management capabilities may have provided these individuals with a sense of security and safety despite the mandatory social isolation [31]. Similar to this study, Chen et al. [19] conducted a survey of people subjected to mandatory social isolation in Guangzhou, China, during the pandemic and found that the majority of the participants did not exhibit symptoms of depression. That said, although the findings of the present study seem inconsistent with those of the previous studies [32], all results essentially verify that adequate and effective public health services are associated with low levels of depression.

This study revealed the risk perception factors associated with depression symptoms among people under home or centralized mandatory isolation. Perceived susceptibility, which pertains to the fear and worry of being infected, could be described as a haunted feeling during social isolation, particularly for those with physical symptoms that may be related to COVID-19 [2]. Meanwhile, under mandatory social isolation, information disseminated through media coverage largely influenced people’s perceptions. Thus, the perceived positive or negative tone of media coverage could establish an overall picture of the fight against the pandemic, thereby enhancing or undermining confidence in the successful response to COVID-19.

People-oriented public health service was identified as an important moderating factor for mental health. Previous studies have emphasized public health services during epidemics and their roles in providing instrumental and informational support for people under mandatory social isolation [5, 33]. Although public health services in most countries may become overwhelmed during the epidemic, the need to maintain high-quality people-oriented services continues to reduce public panic and increase public trust. When people feel understood and cared for by others, they feel less frustrated particularly because public health service providers are representative of local governments and public health management departments [34]. Importantly, people-oriented services enable people under mandatory social isolation to actively cope with the situation and weaken the perceived stigma and xenophobia associated with becoming a potential source of COVID-19 infection [2].

This study has certain theoretical and practical implications. People-oriented services can enhance personal coping strategies and reduce negative emotions during the fight against the pandemic [35]. In this study, the concept of people-oriented public health services during pandemics extends the concept of people-centered medical services to the emergency [36]. People-oriented public health services can be used as a complementary measure for improving the effectiveness of treatment and reducing potential mental health burdens. In addition, these services are typically provided by workers at the community level. Therefore, public health emergency preparedness should include relevant training to address the mental health of the public and prevent diseases simultaneously and to further practice people-oriented public health services in the future [37].

The study has several advantages. First, in the early stages of the pandemic, where people are mainly concerned with the response, prevention, and control efforts exerted by the government, the study focused on the mental health issues of people under mandatory social isolation and investigated their depressive symptoms. Second, the study emphasized the correlation between people-centered public health services and positive perceptions, which was also associated with low levels of depression. Third, the study focused on the interaction between the elements of interpersonal communication and factors of mass communication. Furthermore, the study controlled for many potential influencing factors when exploring mental health during the health crisis. These considerations have built a relatively comprehensive model that can be used to evaluate mental health during public health emergencies.

### Limitations

Despite its strengths, this study has its limitations. First, the random sampling method cannot be used because of the shortage of manpower and resources during the peak time of the pandemic [20]. Therefore, this study conducted a convenience sampling method, constraining its representativeness and generalizability. Individuals with severe mental health problems may also have refused to answer the survey. Thus, the mental health problems of people under mandatory social isolation may have been underestimated. Future studies with sufficient resources can consider using random sampling and door-to-door methods to capture the status of individual mental health problems during epidemics. With the advancement of technology and the Internet of things, other unobtrusive methods (e.g., wearable devices and machine learning techniques) may also assist in identifying individuals with severe mental health problems [38]. Second, the study used a cross-sectional survey; thus, the findings should be interpreted with caution. The temporal sequence between people-centered public health services and depression cannot be determined. Some of the associations may be reversed, and the causal relationships cannot be guaranteed using this type of study design. Hence, future studies using longitudinal surveys or experimental methods are necessary to further explore the causal relationships between mental health and health crises. Third, the researchers were unable to assess the mental health conditions of the participants prior to the survey. Therefore, whether or how their mental health status before the quarantine influenced their current depressive symptoms cannot be determined. In addition, endogenous problems may occur because the measurements were self-reported, and the influence of other factors cannot be excluded. Finally, the study examined the symptoms of depression during the period of social isolation. Future research can further explore the correlates of mental health status post-quarantine or post-pandemic.

## Conclusions

This study examined the depressive symptoms among people under mandatory home or centralized social isolation in Shenzhen, China. The results emphasized the moderating role of people-oriented public health services in the associations among perceived susceptibility, perceived severity, perceived tone of media coverage, and depressive symptoms during the COVID-19 pandemic. Despite the pressure to fight the pandemic, people-oriented public health services for people under mandatory social isolation should be promoted.

## Supplementary Information

**Additional files 1.** Survey questionnaire.

## Data Availability

The datasets used and analyzed during the current study are available from the corresponding author on reasonable request.

## Abbreviations

WHO: World Health Organization
PHQ-9: Patient Health Questionnaire Depression scale
PHS: Public health service
PSU: Perceived susceptibility
PSE: Perceived severity
PTM: Perceived tones of media coverage
PPH: People-oriented public health services
