# Supplementary material for: Moderating effect of people-oriented public health services on depression among people under mandatory social isolation during the COVID-19 pandemic: a cross-sectional study in China
Source: BMC Public Health. 2021 Jul 12;21:1374. doi: 10.1186/s12889-021-11457-6 (PMC8272985; doi:10.1186/s12889-021-11457-6)
Supplement: Supplementary file 1 — Additional files 1. Survey questionnaire. [file 12889_2021_11457_MOESM1_ESM.docx]

**Public health service and mental health survey among people under mandatory social isolation during the COVID-19 pandemic in Shenzhen**

**Online Consent Form**

Dear citizens,

Thank you very much for your cooperation in the prevention and control of the COVID-19 pandemic.

To provide citizens under mandatory social isolation with better support and assistance, Shenzhen Center for Disease Control and Prevention and Shenzhen University’s School of Media and Communication are investigating the public health services that you received and your mental health conditions during the isolation.

This questionnaire is expected to take you about 5 minutes. Participation in the survey is completely anonymous and voluntary. Your information will be kept strictly confidential. There is nothing right or wrong with all the questions, please answer them according to your actual situation and feelings. If at any point in the study you do not want to answer a question or no longer want to participate, you can stop and withdraw from this study without penalty.

Should you have any concerns with the survey, please feel free to contact Shenzhen Center for Disease Control and Prevention or by email to [caobolin@szu.edu.cn](mailto:caobolin@szu.edu.cn). We appreciate your help and best wishes for your health and well-being!

If you understand and agree to participate in this research study, please select “Agree” from the options below. You will then be able to proceed to the questionnaire. We thank you for your participation!

○ Agree

○ Decline (Skip to End of Survey)

**Q. Basic Information (Eligibility Survey) (Q1-2)**

1. Please select your age: ___________

(Questionnaire terminates if participants are under 16 years old)

1. Have you ever been or are currently being mandatory social isolated?

| ○Yes |
| --- |
| ○ No (Questionnaire terminates if this option is selected) |

**Survey Access**

**Part One. Public health service and mental health**

3. Please input the start date of your mandatory social isolation below:

_________________________________

4. Please input the (expected) end date of your mandatory social isolation below:

_________________________________

5. Please select the location of your mandatory social isolation: (Multiple choices)

| □ Home |
| --- |
| □ Hotel |
| □ Others____ |

6. How possible do you think that you could contract the COVID-19 virus during your mandatory social isolation?

| ○ Highly likely |
| --- |
| ○ Somewhat likely |
| ○ Neutral |
| ○ Somewhat unlikely |
| ○ Highly unlikely |

7. Do you have family members who have been confirmed to be infected with COVID-19 virus?

| ○ Yes, your relationship with him/her is ______ |
| --- |
| ○ No |

8. Do you have family members who have been or are undergoing mandatory social isolation?

| ○ Yes |
| --- |
| ○ No |

9. How possible do you think that your family members could contract the COVID-19 virus during mandatory social isolation?

| ○ Highly likely |
| --- |
| ○ Somewhat likely |
| ○ Neutral |
| ○ Somewhat unlikely |
| ○ Highly unlikely |

10. To what extent do you agree with the following statements? Please select the option that best describes your experience and thoughts during the mandatory social isolation.

|  | Strongly disagree | Disagree | Undecided | Agree | Strongly agree |
| --- | --- | --- | --- | --- | --- |
| Public health service workers responded to my question in ways that I can understand. | ○ | ○ | ○ | ○ | ○ |
| Public health service workers cared about my feelings and emotions. | ○ | ○ | ○ | ○ | ○ |
| I perceived the public health service workers as trustworthy | ○ | ○ | ○ | ○ | ○ |

11. During the mandatory social isolation, how often have you been bothered by any of the following problems?

|  | not at all | Several days | More than half the days | Nearly every day |
| --- | --- | --- | --- | --- |
| Little interest or pleasure in doing things | ○ | ○ | ○ | ○ |
| Feeling down, depressed, or hopeless | ○ | ○ | ○ | ○ |
| Trouble falling or staying asleep, or sleeping too much | ○ | ○ | ○ | ○ |
| Feeling tired or having little energy | ○ | ○ | ○ | ○ |
| .Poor appetite or overeating | ○ | ○ | ○ | ○ |
| Feeling bad about yourself or that you are a failure or have let yourself or your family down | ○ | ○ | ○ | ○ |
| Trouble concentrating on things, such as reading the newspaper or watching television | ○ | ○ | ○ | ○ |
| Moving or speaking so slowly that other people could have noticed. Or the opposite being so figety or restless that you have been moving around a lot more than usual | ○ | ○ | ○ | ○ |
| Thoughts that you would be better off dead, or of hurting yourself | ○ | ○ | ○ | ○ |

12. For how long do you think COVID-19 would continue to affect your life?

| ○ Less than 1 week |
| --- |
| ○ Between 2 to 4 weeks |
| ○ Between 1 to 2 months |
| ○ Between 3 to 5 months |
| ○ More than 6 months |
| ○Don’t know |

**Part Two: Media use and social support**

13. How much time do you spend on COVID-19 news and information? (Including reading news on a cell phone, watching television news, and talking to family and friends)?

| ○ Less than one hour |
| --- |
| ○ 1-2 hours |
| ○ 3-4 hours |
| ○ 5-6 hours |
| ○ More than 7 hours |

14. How would you rate the tone of media coverage of the COVID-19 pandemic? Based on a bipolar scale, please select the appropriate option.

| Negative | -3 -2 -1 0 1 2 3 | Positive |
| --- | --- | --- |
| Critical | -3 -2 -1 0 1 2 3 | Encouraging |
| Complaining | -3 -2 -1 0 1 2 3 | Forgiving |
| Nonreflective | -3 -2 -1 0 1 2 3 | Reflective |
| Worried | -3 -2 -1 0 1 2 3 | Composed |
| Indifferent | -3 -2 -1 0 1 2 3 | Touching |
| Timid | -3 -2 -1 0 1 2 3 | Brave |

15. Please select the isolation status that best fits your situation:

| ○ Living with family/ friends during isolation |
| --- |
| ○ Living away from family/ friends during isolation |
| ○ Other situations______________ |

16. Please indicate how likely you are to agree with the following statements during the mandatory social isolation.

|  | Not like me at all | Somewhat not like me | Neutral | Somewhat like me | Very like me |
| --- | --- | --- | --- | --- | --- |
| When I feel scared, I turn online to my relatives and/or friends to talk about my feelings. | ○ | ○ | ○ | ○ | ○ |
| When I feel lonely and afraid, I confide in strangers through the Internet. | ○ | ○ | ○ | ○ | ○ |
| When friends and family members worry about me, I calm them down online. | ○ | ○ | ○ | ○ | ○ |
| I have learned a lot about the symptoms and information related to COVID-19 pandemic online. | ○ | ○ | ○ | ○ | ○ |
| I have discussed with others online about things to be watched out during the mandatory social isolation. | ○ | ○ | ○ | ○ | ○ |
| I have tried to get help from strangers online. | ○ | ○ | ○ | ○ | ○ |
| My request has been answered by someone else online. | ○ | ○ | ○ | ○ | ○ |
| I have offered advice to others online about how to take care of themselves. | ○ | ○ | ○ | ○ | ○ |
| Strangers online have offered me practical help (e.g., money donations) | ○ | ○ | ○ | ○ | ○ |

17. Please indicate how likely you are to agree with the following statements during the mandatory social isolation.

|  | Not like me at all | Somewhat not like me | Neutral | Somewhat like me | Very like me |
| --- | --- | --- | --- | --- | --- |
| I have found a couple of people in a similar situation with me on the Internet. | ○ | ○ | ○ | ○ | ○ |
| I have joined groups of people in similar situations online. | ○ | ○ | ○ | ○ | ○ |
| I have shared the information I know related to COVID-19 in online groups. | ○ | ○ | ○ | ○ | ○ |
| I have posted information about mandatory social isolation on social media. | ○ | ○ | ○ | ○ | ○ |
| I have received a lot encouragements after I posted information related to my mandatory social isolation on social media. | ○ | ○ | ○ | ○ | ○ |
| My friends have asked me if I need any instrumental help online (e.g., money donation) | ○ | ○ | ○ | ○ | ○ |
| The Internet has allowed me to enjoy my life as before during the isolation. | ○ | ○ | ○ | ○ | ○ |
| The online interactions have made me feel cared by a lot of people during the period. | ○ | ○ | ○ | ○ | ○ |
| I have cared for others through the Internet during the isolation, hoping them to live a better life in the future. | ○ | ○ | ○ | ○ | ○ |

**Part Three. Demographics**

18. Your sex:

| ○ Male |
| --- |
| ○ Female |

19. Your education:

| ○ Junior high school or below |
| --- |
| ○ High school |
| ○ Undergraduate |
| ○ Master or above |

20. Your monthly income:

| ○ No income |
| --- |
| ○ Lower than 5000 RMB |
| ○ 5000-8000 RMB |
| ○ 8001-12000 RMB |
| ○ 12001-30000 RMB  21. Which district do you live:  ○ Luohu District  ○ Longgang District |

**Thanks for participating our survey!**
